# Effects of manual lymphatic drainage on total knee replacement: a systematic review and meta-analysis of randomized controlled trials

**DOI:** 10.1186/s12891-023-07153-8

**Published:** 2024-01-02

**Authors:** Hongyuan Lu, Quanwei Shao, Wenyao Li, Fei Li, Weiyi Xiong, Kunpeng Li, Wei Feng

**Affiliations:** 1 Guanghua Integrated Traditional Chinese and Western Medicine Hospital, Shanghai, 200052 China; 2 Yangpu District Central Hospital, Shanghai, 200090 China; 3 Shanghai No.4 Rehabilitation Hospital, Shanghai, 200040 China; 4 School of Rehabilitation Science, Shanghai University of Traditional Chinese Medicine, Shanghai, 201203 China; 5 Shanghai Fourth People’s Hospital, Shanghai, 200040 China; 6 The Second Rehabilitation Hospital of Shanghai, Shanghai, 202441 China

**Keywords:** Manual lymphatic drainage, Total knee replacement, Edema, Range of motion, Pain

## Abstract

**Background:**

Total knee joint replacement (TKR) is an effective method for the treatment of severe knee osteoarthritis. With an increasing number of surgeries, complications such as lower limb edema, pain, and limited mobility have caused a heavy burden. Manual lymphatic drainage (MLD) may be a solution to solve the problem. The study aims to evaluate the efficacy of MLD in reducing knee edema, pain, and improving range of motion (ROM) in patients after TKR.

**Methods:**

A search was conducted in PubMed, Embase, Cochrane Library, Web of Science, CNKI, VIPs, WanFang database, and Google Scholar from inception to June 2023. Only randomized controlled trials (RCTs) that compared the effects of MLD and non-MLD (or another physiotherapy) on improving knee edema, pain, and ROM after TKR were included. Stata 16.0 was used for meta-analysis. GRADE was used to assess the quality of evidence.

**Results:**

In total, 7 RCTs with 285 patients were identified. There were no significant differences found in the ROM of knee flexion (standardized mean difference (SMD) = 0.03, 95% confidence interval (CI): -0.22, 0.28, *P* = 0.812) and the ROM of knee extension (SMD= -0.30, 95%CI: -0.64, 0.04, *P* = 0.084). No differences were observed in the lower extremity circumference after TKR (SMD= -0.09, 95%CI: -0.27, 0.09, *P* = 0.324). For postoperative pain, there was no significant advantage between the MLD and non-MLD groups (SMD= -0.33, 95%CI: -0.71, 0.04, *P* = 0.083).

**Conclusions:**

Based on the current evidence from RCTs, manual lymphatic drainage is not recommended for the rehabilitation of patients following total knee replacement.

**Supplementary Information:**

The online version contains supplementary material available at 10.1186/s12891-023-07153-8.

## Introduction

Osteoarthritis of the knee is a prominent factor contributing to disability among adults aged 65 years old and over [1]. Progressive joint destruction is caused by osteoarthritis, which impacts all anatomical components of the joint [2]. End-stage knee osteoarthritis can be effectively treated with total knee replacement (TKR) [3]. An estimated 700,000 TKR are performed annually in the United States, with projections indicating that the number will increase to 3.5 million per year by 2030 [4]. The efficacy and affordability of TKR have been demonstrated in clinical studies [5]. However, a considerable proportion of individuals express dissatisfaction with the outcomes of TKR [6]. Firstly, untreated chronic swelling could exacerbate discomfort and lead to difficulties in mobility and flexibility [7]. Moreover, edema potentially increases the risk of infection in the impacted region, reduces the flow of blood, and impacts the elasticity of the arteries [8]. Individuals are greatly affected by these complaints, causing both physical and psychological harm.

Typically, it is advised to undergo some type of early rehabilitation (within 0–6 weeks) prior to being discharged from the hospital. Physiotherapy following TKR involves the utilization of techniques to alleviate pain and edema, targeted physical activities to enhance the flexibility of the joint, and exercises to improve muscle strength and endurance [9, 10]. Nevertheless, the current evidence for the use of early rehabilitation after TKR is limited, and some studies indicated that certain techniques like cryotherapy, compression, and pulsed electromagnetic fields have shown no impact on knee swelling [11, 12].

Manual lymphatic drainage (MLD) involves specialized rhythmic pumping techniques employed to massage the affected region and improve the flow of lymph [13]. MLD has the potential to decrease swelling by encouraging the pumping action of lymphangion and redirecting lymph away from stagnant areas to functional lymphatic vessels by reducing hydrostatic pressure [14]. Some studies reported the effectiveness of MLD in decreasing lymphatic swelling in females related to breast cancer [15, 16]. The application of MLD in musculoskeletal diseases has also garnered significant interest. Nevertheless, there are still some disputes regarding the effectiveness of MLD in patients receiving TKR. It has been shown in several randomized controlled trials (RCTs) that MLD, in combination with standard rehabilitation protocols, has positive effects on early edema and pain levels [17–19]. However, it has also been reported in several articles that the application of MLD in the early postoperative period after TKR does not reduce swelling [12, 20]. Can MLD be a routine therapy added following TKR or just be applied when the appearance of lower limb edema or pain? Despite the reporting of several RCTs that applied additional MLD after the surgery, there has been a lack of relevant meta-analysis and systematic review. An immediate study is necessary to assess whether MLD is more effective than standard routine rehabilitation or another physiotherapy in improving the following aspects: (1) lower limb circumferences or volume; (2) range of motion (ROM) of the knee joint; (3) knee joint pain.

## Methods

The study was performed following relevant requirements suggested by the Cochrane Handbook [21] and was reported following the guidelines of the Preferred Reporting Items for Systematic Reviews and Meta-Analysis (PRISMA) statement [22]. The protocol has been registered on PROSPERO: CRD42023441423.

### Search strategy

PubMed, Embase, Cochrane Library, Web of Science, CNKI, VIPs, WanFang database, and Google Scholar were utilized for the search from inception to June 2023. Specific databases were customized with tailored keywords and corresponding Medical Subject Headings (MeSH) terms for “Manual Lymphatic Drainage” AND “Arthroplasty, Replacement, Knee”. All search terms were utilized due to the interchangeable use of different terms in the literature including TKR, TKA, knee arthroplasty, manual lymph drainage, decongestive lymphatic therapy, and Foldi or Vodder method. The detailed search strategy can be found in supplementary file 1. Additional eligible studies were manually examined by referring to reference lists obtained from retrieved articles. After removing duplicates, the titles and abstracts were reviewed independently by two authors (HYL and QWS) based on the inclusion criteria. In July 2023, the search was repeated in the same databases to identify any new trials or previously overlooked studies. Since MLD is mostly used for the treatment of postoperative lymphedema of breast cancer, such trials related to TKR were fewer, other resources were searched but were not limited to (1) monthly search of the Cochrane Controlled Trial Center Register (CENTRAL); (2) manual search of MLD related journals and major TKR conference documents; (3) contact researchers who are known to have participated in previous experiments to seek information about unpublished or incomplete experiments; (4) reference lists for review articles and related experiments in case of any possible omission.

### Inclusion and exclusion criteria

#### Inclusion criteria

(1) Population: patients who underwent TKA/TKR; (2) Intervention: application of MLD; (3) Comparison: no application of MLD or other physical therapy; (4) At least one of the following outcomes: knee edema (knee circumferences or volume); ROM of the knee joint; pain and knee function. Only studies defined MLD as the application of a gentle massage technique by trained therapists, following the lymphatic pathways in a certain direction (from proximal to distal and then following the opposite way [23] or claimed to have followed the ‘Foldi or Vodder method’ were included. To meet the inclusion criteria, MLD needed to be employed as the primary intervention, not as a co-intervention with other physical therapy treatments in studies. In addition, if a study designed a comprehensive rehabilitation program, MLD had to possess over 50% of the program as the main treatment to be included.

The outcome measures included knee edema (knee circumferences or volume), ROM of the knee joint, and pain [using the Visual Analogue Scale (VAS), McGill Pain Questionnaire, and Numerical Rating Scale (NRS)]. For knee function, Knee Injury and Osteoarthritis Outcome Score (KOOS), EuroQol Five Dimensions Questionnaire (EQ-5D), Functional independence measurement (FIM), and Knee Society Score (KSS) were extracted.

#### Exclusion criteria

(1) Reviews, meetings, and case reports; (2) Duplicate publications or studies with similar data; (3) Data that is incomplete, ambiguous, or unable to be extracted or compared.

### Study selection

Two researchers (HYL and QWS) conducted separate searches of the databases and examined the titles and abstracts of the studies that were retrieved. Any ambiguities between the reviewers (HYL and QWS) were bought and discussed with the third reviewer (KPL). The eligible studies under the inclusion and exclusion criteria were carried out after reading the complete text.

### Data extraction

HYL and QWS performed data extraction, and any disagreements were settled by KPL. Data extraction included: first author, publication year, country, sample size, the average age of control and experimental patients, diagnosis, study type, frequency and intervention time of control and experimental group, outcome assessments, and follow-up.

### Risk of bias assessment

The risk of bias assessment was evaluated by two independent reviewers (HYL and QWS) respectively. If there were any disagreements to reach the final assessment, a third author (KPL) was consulted. Regarding RCTs, the Cochrane Risk of Bias tool ver.2 for RCTs (Cochrane RoB 2 tool) was employed [24]. Five domains of bias were assessed: (1) bias in selection; (2) bias in performance; (3) bias in attrition; (4) bias in detection; and (5) bias in selective outcome reporting [24, 25]. The Grading of Recommendations, Assessment, Development, and Evaluations approach (GRADE) was employed to assess the certainty of the evidence [26].

### Statistical analysis

Stata 16.0 (STATA Corporation, Lakeway, Texas, USA) was utilized for all statistical analyses. Standardized mean differences (SMD) with 95% CIs for continuous outcomes were pooled. The I^2^ statistic and Q test (χ^2^) were used to quantify the heterogeneity of included studies, with a significance level of *P* ≤ 0.050 [27]. Based on the analysis of I^2^ values, heterogeneity may not be important: 0–40%; moderate heterogeneity: 30–60%; substantial heterogeneity: 50–90%, and considerable heterogeneity: 75–100% [21]. To account for clinical heterogeneity, a random-effects model was employed to combine outcome data. If possible to check various factors affecting MLD, a subgroup analysis may be performed according to (1) the time point and dosage for starting MLD after surgery; (2) different control interventions (other forms of exercises, kinesio tape, mixed physical therapy techniques or no intervention). Separate meta-analyses should be performed for each subgroup.

Given the inadequate quantity of research conducted, publication bias was evaluated through the utilization of a funnel plot and further assessed using the linear regression tests of Egger and Begg [28]. A sensitivity analysis is presented. Besides, there were reports of potential conflicts of interest and disclosure of the funding source.

## Results

### Literature search

**Fig. 1:**
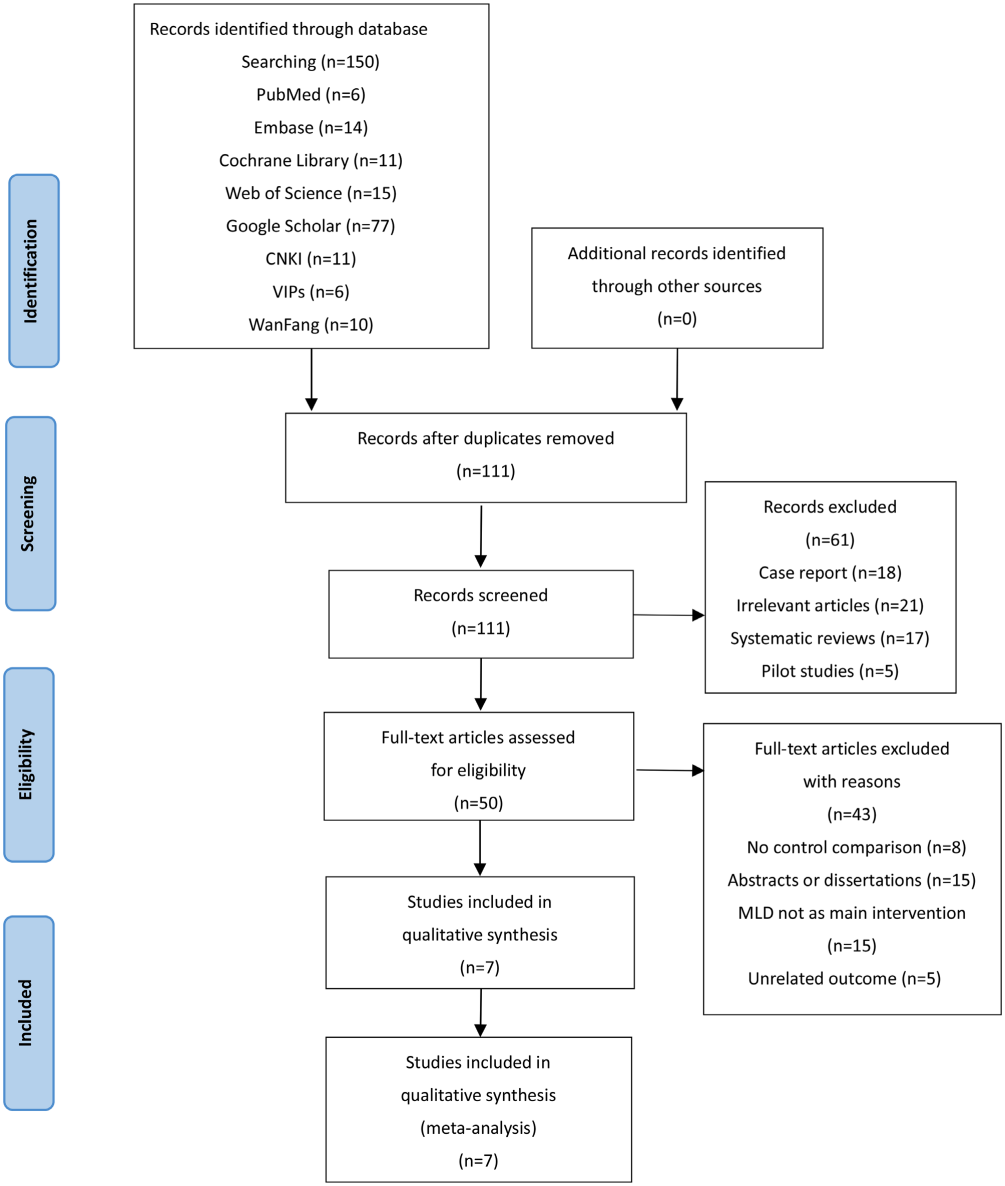
The flow diagram of study selection

The detailed literature screening process is shown in Fig. 1. A total of 150 articles were retrieved, after removing duplicates from 39 citations, there were 111 articles remaining. Then, 61 studies including 18 case reports, 21 irrelevant articles, 17 systematic reviews, and 5 pilot studies were excluded. After that, 50 studies were thought to be potentially eligible and 43 articles were excluded for reasons. Finally, 7 RCTs from 5 countries were confirmed after full-text review and included in the systematic review and meta-analysis.

### Study characteristics

The basic characteristics of the included studies are summarized in Table 1. Among the 7 included trials, 3 studies were conducted in Turkey [12, 17, 18] and one each in Switzerland [20], Australia [29], Japan [30], and Italy [31]. The included trials were published in English between 2013 and 2022, with a total of 285 patients enrolled. There were 6 of 7 trials that compared MLD with non-MLD and one trial compared MLD with kinesiotape. The age of the patients ranged from 48 to 89, with an average age (standard deviation) of 54.38 (9.81). 5 of 7 studies included 228 patients (80%) who had a primary diagnosis of knee osteoarthritis before the surgery while 57 patients of 2 studies had unclear diagnoses before surgery. All patients in the included studies underwent TKR/TKA by an experienced orthopedic surgeon. In all 7 RCTs, MLD treatment was initiated 2 days after surgery. The duration of the MLD treatment course was reported as 2–4 days postoperative in 2 studies (28.6%), over 6 days of treatment in 2 studies (28.6%), the second day postoperative in one study (14.3%), and the second and fourth day postoperative in 2 studies (28.6%).

**Table 1:** General characteristics of the included studies

First Author, Year	Country of study	Intervention group(Sample Size)	Control group(Sample Size)	Intervention group	Control group	Intervention time	Follow up time	Outcomes measures
Vergili et al., 2022 [12]	Turkey	8	8	MLD + physical therapy	NO MLD + physical therapy	30 min/day on the second and fourth day after surgery	D2, D4 and D6 after surgery	ROM; pain:VAS; edema: knee volume; function: FIM
CiHAN et al., 2021 [17]	Turkey	11	11	MLD + physical therapy	NO MLD + physical therapy	No technical details	D3 and 6 weeks after surgery	pain:VAS; function:NHP; fear:TKS
Guney-Deniz et al., 2022 [18]	Turkey	13	15	MLD + exercises	NO MLD + exercises	30 min/day, 2–4 days after surgery	D2, D3, D4, 2 weeks and 6 weeks after surgery	ROM; pain:VAS; function:KOOS; edema:knee circumference
Pichonnaz et al., 2016 [20]	Switzerland	29	27	MLD + physical therapy	NO MLD + physical therapy	30 min/day, 2–7 days after surgery	D2, D7, and 3 months after surgery	ROM; pain:VAS; function:KSS,WOMAC; edema:BIS limb volume
Ebert et al., 2013 [29]	Australia	24	26	MLD + physical therapy	NO MLD + physical therapy	30 min/day, 2–4 days after surgery	D2, D3, D4, and 6 weeks after surgery	ROM; pain:NRS; function:KOOS; edema:knee girths
Fujiura et al., 2020 [30]	Japan	20	20	MLD + physical therapy	NO MLD + physical therapy	20 min/day, 2–6 days after surgery	Immediately and the fifth MLD application after surgery	ROM; pain:VAS; edema:knee circumference
Tornatore et al., 2020 [31]	Italy	33	33	MLD + education	Kinesio tape + education	30 min/day on the second and fourth day after surgery	D4, D6 after surgery	ROM; pain:NRS; edema:knee circumference

Note: MLD: Manual lymphatic drainage; VAS: Visual Analogue Scale; NRS: Numeric Rating Scale; FIM: Functional independence measurement; TKS: Tampa Kinesiophobia Scale; KOOS: Knee Injury and Osteoarthritis Outcome Score; NHP: Nottingham Health Profile; BIS: Bioimpedance Spectroscopy; WOMAC: Western Ontario and McMaster Universities Osteoarthritis Index; KSS: Knee Society Score; D: Day

### Risk of bias assessment

**Fig. 2:**
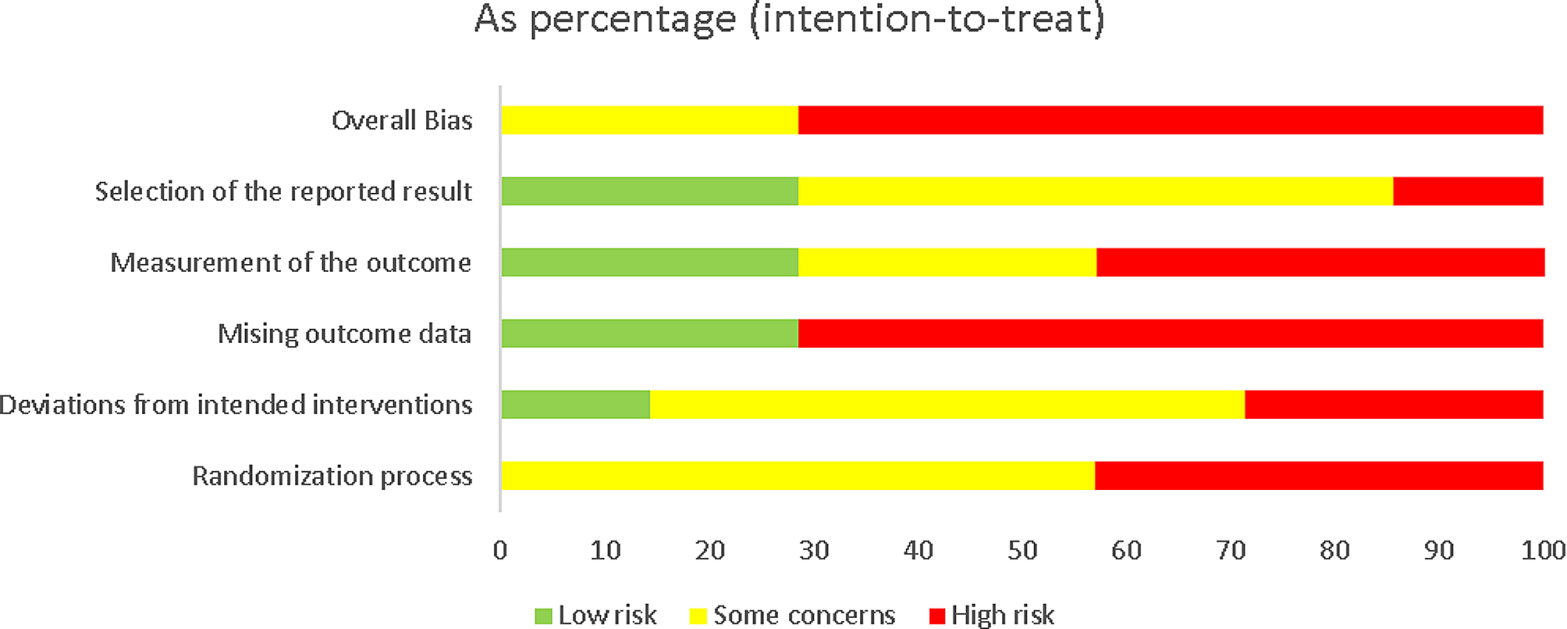
Risk of bias graph showing each risk of bias item as percentages across all included studies

**Fig. 3:**
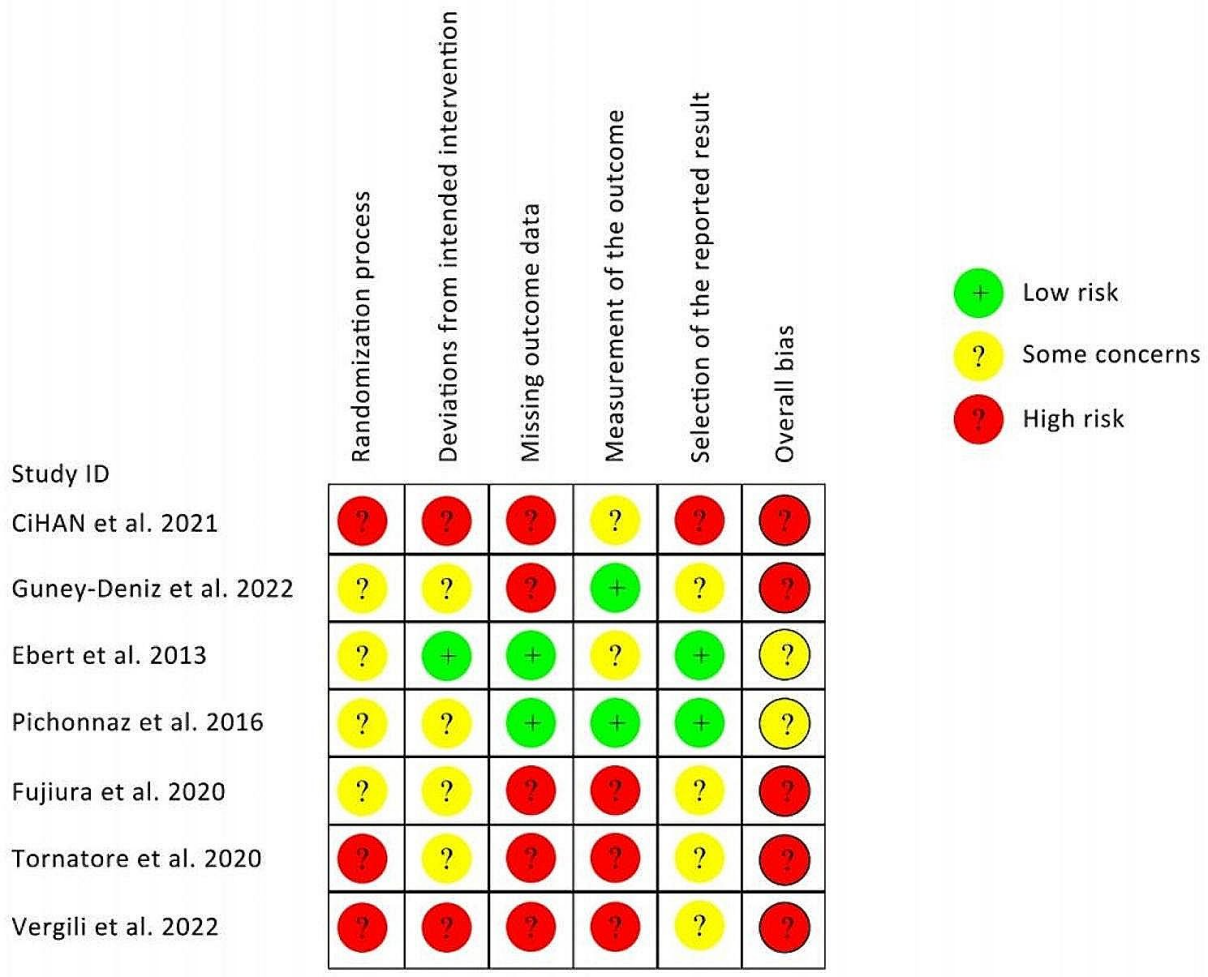
Risk of bias summary for included studies

A summary of the risk of bias assessment is presented in Fig. 2. Figure 3 provides the bias risk for each study included, as well as for each domain. In general, most of the studies (71%; n = 5) were judged as ‘high risk of bias’ whereas two studies (29%) were considered to have ‘some concern’. An inadequate randomized sequence and allocation concealment of three studies were considered to be ‘high risk’. The majority of studies (71%) failed to sufficiently balance baseline covariates among the groups. Five studies did not report blinding of outcome assessments or provide information about its absence. In addition, only three studies (42.8%) registered their protocol following a prespecified analysis plan. Refer to supplementary file 2 for detailed information regarding the quality of evidence for the measured outcomes.

### Funding and conflicts of interest

Two studies were funded by university or hospital research foundations [20, 29]. Two studies [12, 18] did not receive any forms of foundations while the other three studies were unclear [17, 30, 31]. The authors of six studies [12, 18, 20, 29–31] declared no conflicts of interest, and one study [17] had no official disclosure of conflicts of interest report.

### Effects of interventions

#### ROM of the knee joint

**Fig. 4:**
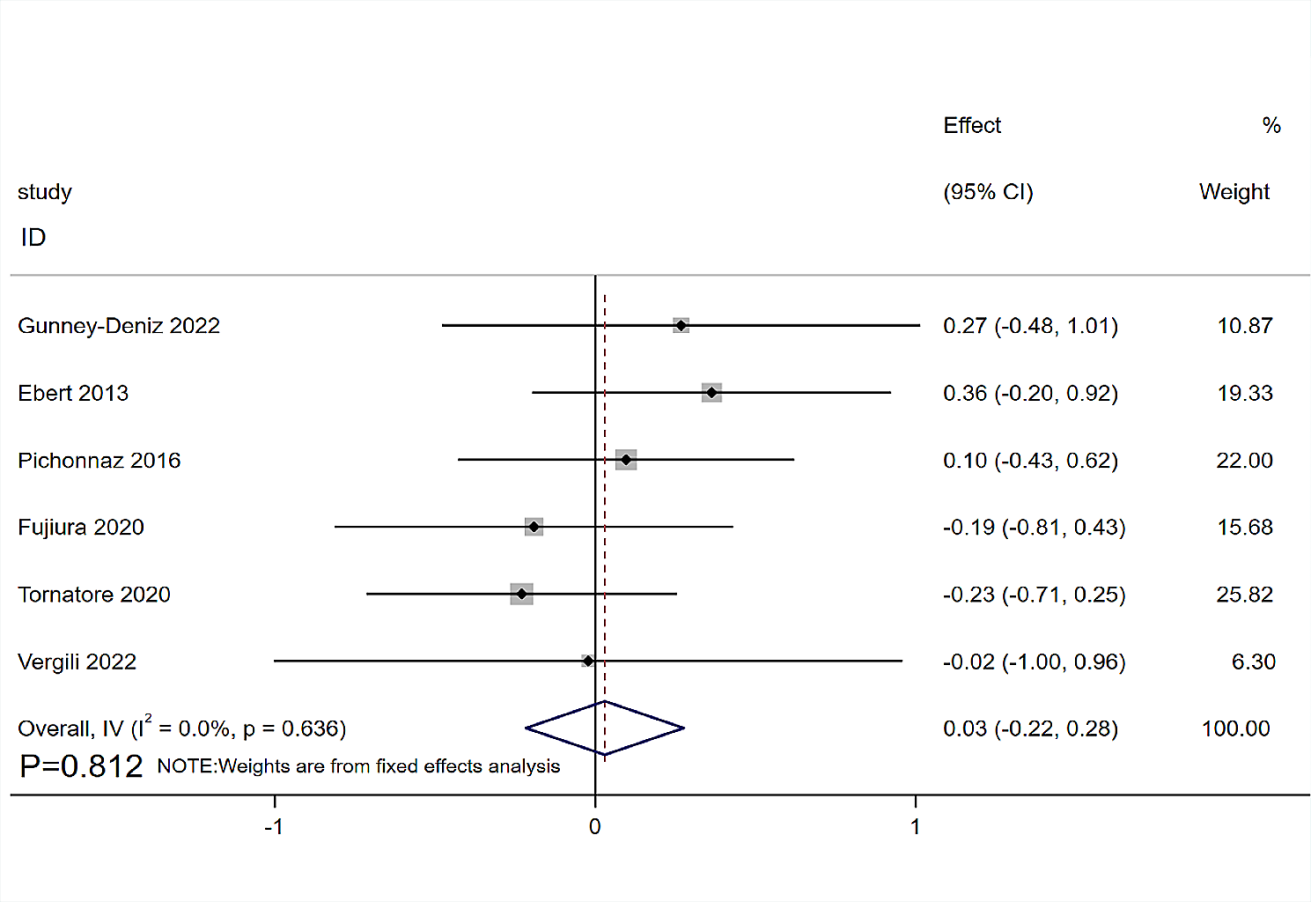
Forest plot of knee flexion range of motion

**Fig. 5:**
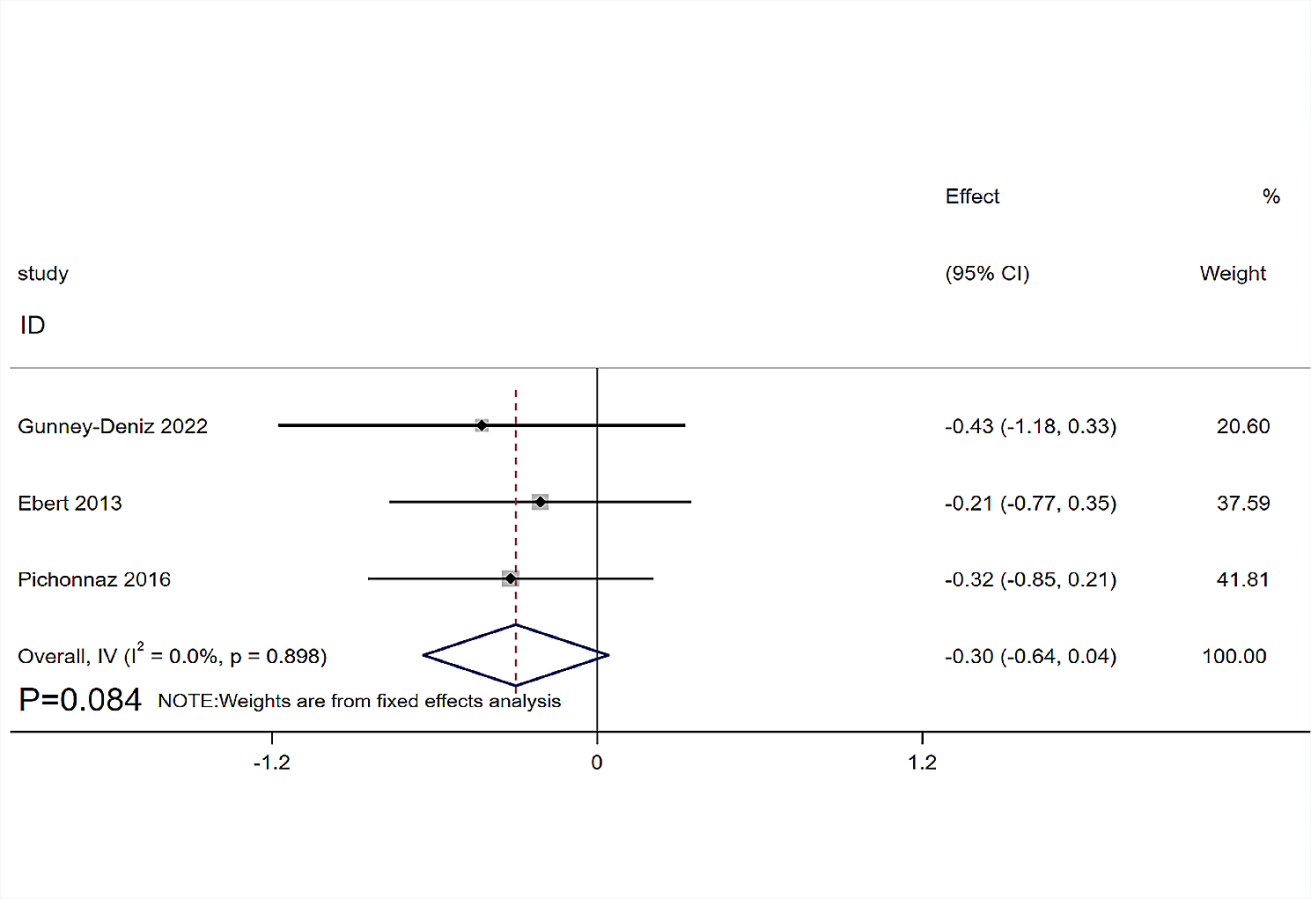
Forest plot of knee extension range of motion

Six RCTs [12, 18, 20, 29–31] involving 256 patients provided data on the ROM of the knee joint. The follow-up time is mostly concentrated on the fourth day after the operation [12, 18, 29, 31], and the seventh day in one RCT [20]. Six RCTs [12, 18, 20, 29–31] compared the ROM of knee joint flexion, and three RCTs [18, 20, 29] compared the ROM of knee joint extension. The heterogeneity was low (I^2^ = 0%, *P* = 0.636), which was analyzed using a fixed-effects model. The MLD therapy demonstrated no significant advantage over non-MLD groups both in knee flexion (SMD = 0.03, 95%CI: -0.22, 0.28, *P* = 0.812, Fig. 4) and knee extension (SMD= -0.30, 95%CI: -0.64, 0.04, *P* = 0.084, Fig. 5).

#### Pain after Surgery

**Fig. 6:**
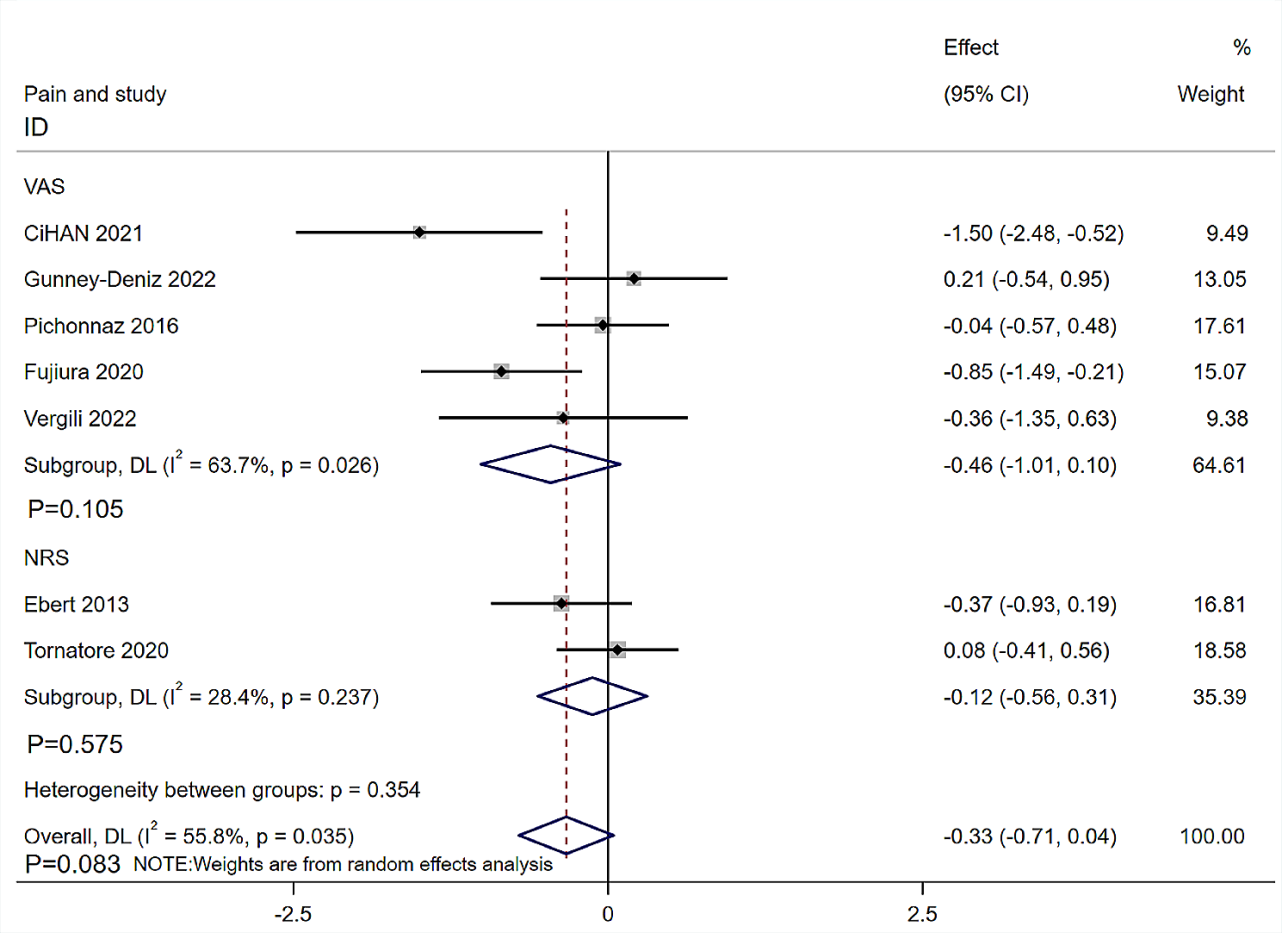
Forest plot of knee joint pain assessed by VAS and NRS. VAS: Visual Analogue Scale; NRS: Numerical Rating Scale

Seven RCTs [12, 17, 18, 20, 29–31] with 278 patients reported postoperative pain. The follow-up time of four RCTs was the fourth day after TKR [12, 18, 29, 31], the third day after the operation [17], the seventh day after the operation in one study [20], and after the fifth application of MLD in one study [30]. Five RCTs [12, 17, 18, 20, 30] used the Visual Analogue Scale (VAS) and two RCTs [29, 31] used the Numeric Rating Scale (NRS). Subgroup analysis of the knee joint pain was conducted based on different measurements. The heterogeneity was moderate (I^2^ = 55.8%, *P* = 0.035), which was analyzed using a random-effects model. Patients treated with MLD had no benefit in relieving pain after TKR than non-MLD groups (SMD= -0.33, 95%CI: -0.71, 0.04, *P* = 0.083, Fig. 6).

#### Knee edema

**Fig. 7:**
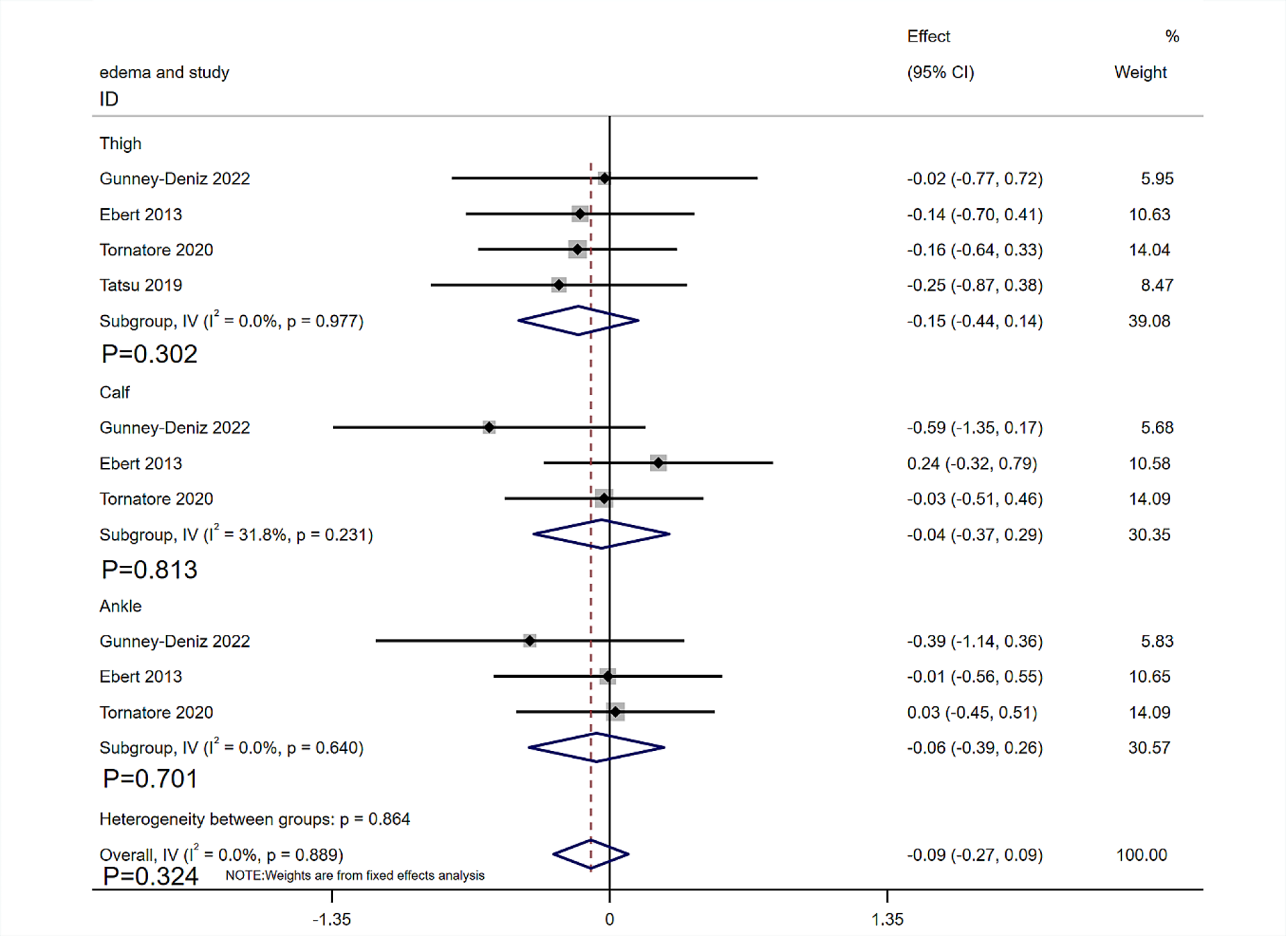
Forest plot of knee edema assessed by thigh, calf, and ankle circumferences

The knee edema measurements included thigh, calf, and ankle circumferences. Four RCTs [18, 29–31] with 184 patients reported lower limb lymphedema. Four RCTs [18, 29–31] provided data on thigh circumferences, and three RCTs [18, 29, 31] provided calf and ankle circumferences. The heterogeneity was low (I^2^ = 0%, *P* = 0.889), which was analyzed using a fixed-effects model. No significant differences were found between the MLD and non-MLD groups in terms of thigh circumferences (SMD= -0.15, 95%CI: -0.44, 0.14, *P* = 0.302), calf circumferences (SMD= -0.04, 95%CI: -0.37, 0.29, *P* = 0.813) and ankle circumferences (SMD= -0.06, 95%CI: -0.39, 0.26, *P* = 0.701). The final results showed that compared with non-MLD groups, MLD did not improve knee edema after TKR (SMD= -0.09, 95%CI: -0.27, 0.09, *P* = 0.324, I^2^ = 0%, Fixed Effect Model, Fig. 7).

#### Evaluation of publication bias

**Fig. 8:**
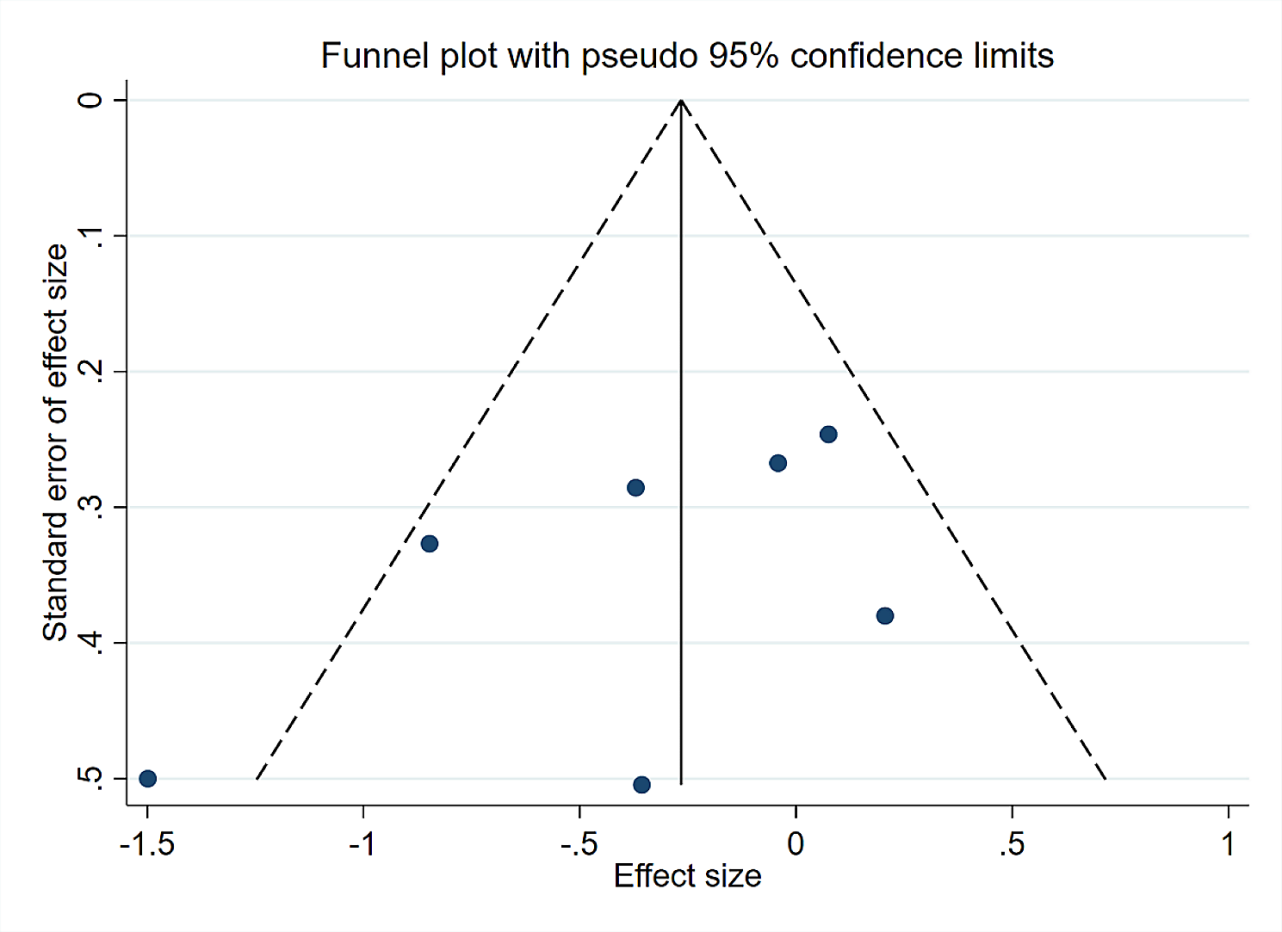
Funnel plot of knee joint pain after TKR surgery. TKR: Total knee replacement

**Fig. 9:**
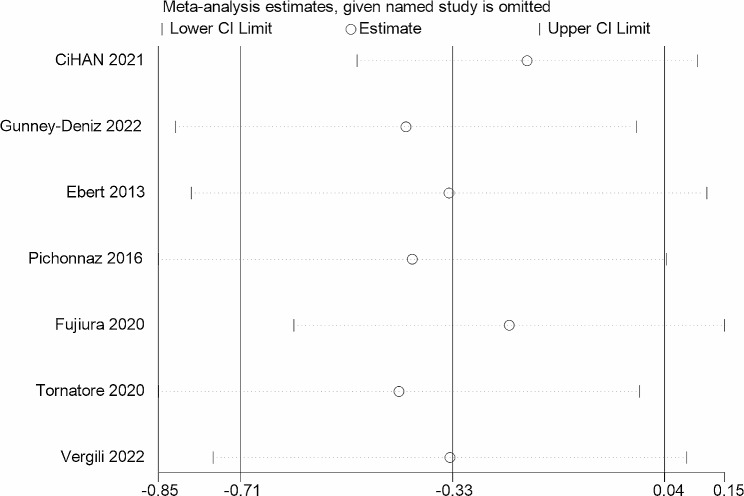
Sensitivity analysis of knee joint pain after TKR surgery. TKR: Total knee replacement

There was no clear evidence of publication bias when examining the funnel plot (Fig. 8) for the impact of MLD compared to non-MLD groups on postoperative pain. The results of Egger’s test showed no publication bias on knee joint pain (*P* = 0.200). Additionally, a sensitivity analysis was conducted to evaluate the stability of the combined findings. The sensitivity analysis indicated that the results were statistically reliable (Fig. 9) after sequentially omitting each study and the outcomes were not altered.

## Discussion

Seven RCTs assessing the efficacy of MLD in patients with pain, limited ROM, and lower limb edema after TKR were included. The findings of the current study indicated that MLD did not demonstrate significant reductions in lower limb edema, pain, or improve joint mobility when compared to the absence of MLD or other physiotherapy interventions after TKR.

Furthermore, previous studies also indicated that early MLD for patients undergoing breast cancer surgery cannot significantly reduce or prevent lymphedema [13, 32, 33]. Additionally, a separate systematic review expressed uncertainty regarding the effectiveness of MLD [34]. However, Provencher AM et al. [35] have provided support for the use of MLD in reducing swelling and pain, improving mobility, and enhancing patient satisfaction and quality of life following musculoskeletal injuries. Gutiérrez-Espinoza H’s systematic review and meta-analysis [36] concluded that the inclusion of mobilization with movement and MLD resulted in significant improvements in the wrist, upper limb function, and hand edema among individuals diagnosed with distal radius fracture. Klein et al. [7] revealed that only the application of multiple layers and prolonged compression effectively decreases swelling following orthopedic injury or surgical procedures. Schingale et al. [37] conducted a narrative review and found that MLD has the potential to be utilized for the symptomatic management of different diseases, including multiple sclerosis and Parkinson’s disease. The potential effectiveness of MLD in animal models was shown in three animal experiments [38–40], but its applicability in humans is currently uncertain.

There is a lack of consensus among published studies regarding the efficacy of MLD therapy. Most studies focused on the application of MLD for treating lymphedema in females following breast cancer surgery. Apart from the negative outcomes indicated in the aforementioned studies [13, 32, 33], Ezzo J et al. [41] included six trials and the findings were contradictory for function (ROM). Regardless of the treatment they received, 60–80% of participants reported improved sensations, including pain relief and reduced heaviness. One RCT revealed that standard therapy and MLD in combination with multilayer compressive bandage treatment were both effective [42]. Nevertheless, there were no further effects observed in terms of a decrease in arm volume percentage during the period of intensive treatment after breast cancer surgery. On the contrary, a systematic review [43] indicated that MLD may yield positive outcomes in terms of reducing volume, enhancing quality of life, and alleviating symptoms following breast cancer surgery. However, the author also indicated that MLD may not provide additional benefits for patients with moderate to severe lymphedema. Doubblestein and colleagues’ review [44] suggested the utilization of MLD to reduce the incidence of lymphedema in early rehabilitation post-surgery. The author included 5 studies (192 patients) focusing on acute musculoskeletal disorders and subacute edema. In contrast with the present systematic review and meta-analysis, the author found that MLD could potentially be beneficial in reducing edema and enhancing ROM when combined with auxiliary therapies. Nevertheless, an absence of ample evidence and high-quality RCTs to endorse the utilization of MLD in enhancing the overall clinical manifestation.

The present review conducted the comparison between MLD and non-MLD (or another physiotherapy), with only one RCT utilizing MLD and kinesiotape for comparison. The effectiveness between MLD and other compression therapy remained unclear. One systematic review and meta-analysis evaluating the effects of MLD and compression therapy found that compression bandaging was effective in managing breast cancer-related lymphedema [13]. One RCT conducted by Rigoni et al. [45] compared MLD and connective tissue techniques for the treatment of post-surgical inflammation in patients with TKA. The findings indicated that both approaches had a beneficial impact on the outcomes and demonstrated comparable effectiveness in regulating the erythrosedimentation rate (ESR) for both methods. Bertinchamp et al. [19] found that both MLD and kinesiotape improved ROM and decreased pain, but MLD demonstrated greater quality of life scores in patients after TKA. Preneuf-Pauthiera et al. [46] concluded that no significant statistical difference was found between MLD and usual physiotherapy regarding ROM, but potential effects on decreasing pain were found in the MLD group. Given the trend of pain reduction, the author considered a large sample and the type of edema (superficial or deep) should be taken into consideration to verify this finding.

The meta-analysis of the results related to knee joint pain showed moderate heterogeneity, while the heterogeneity in the remaining outcomes was relatively low. The heterogeneity observed among the knee joint pain examined may be attributed to differences in the clinical setting and variations in measurement techniques. Firstly, the included studies differed in terms of the duration of MLD usage. Five studies [12, 18, 20, 29, 31] applied MLD lasting 30 min, one study [30] applied MLD lasting 20 min and one study [17] did not specify the technical details of the MLD method. Secondly, the use of MLD was mainly applied between 2 and 6 days while two studies [12, 31] applied MLD on the second and fourth day after the surgery. Finally, the outcomes could be affected by the experience of the physiotherapist or the specific operation of the MLD method. Four studies [12, 18, 29, 30] listed the specific process of the MLD method and tended to be similar. One study [20] claimed that their research was based on the recommendations of Földi and Kubik [47]. While the other one [17] claimed that their MLD was performed by a physiotherapist with a medical certificate. However, another study [31] did not specify the method used for MLD.

### Clinical implications

Swelling following TKR leads to pain, inflammation, limited mobility, hindered quadriceps function, and a negative perception of recovery [48]. The prevention and treatment of initial edema after TKR is very important. The strength of this research lies in the potential benefit of MLD as a singular method to relieve patients’ burdens, since MLD is convenient and economical. The study fills the blank of the meta-analysis and systematic review of the efficacy of MLD for patients receiving TKR. However, the effect sizes did not reach a statistical difference when using MLD compared to not using MLD, which could be attributed to the small sample size of the included studies, with only one RCT involving over 60 participants [31]. Large-scale, high-sample clinical trials in the future may provide valuable evidence to support the widespread application of MLD in clinical practice. Furthermore, studies investigating MLD use alone (without concurrent treatments) were limited [49], making it challenging to obtain conclusive evidence supporting the standalone efficacy of MLD as a swelling reduction technique. In summary, based on the available RCT evidence, there is insufficient support for MLD as a stand-alone edema reduction technique after TKR in terms of reducing lower limb edema, pain and improving ROM of the knee joint.

### Limitations and future scope

Certain limitations should be noted in the current study. Firstly, the total samples of the included studies were small, of which one study only recruited 8 patients in each group [12]. Secondly, only one of the included studies involved kinesiotape as a control group [31], making it difficult to be generalized to other forms of compression therapy. Finally, the follow-up periods in the majority of the studies were relatively short, mainly concentrated within the first week after surgery [12, 30, 31]. To address these limitations, future research is needed to focus on larger sample sizes, more rigorous, and higher-quality RCTs of a single application of MLD. Furthermore, future studies could be conducted to explore the inclusion of other compression techniques in the control group and compare these with MLD. Lastly, further studies are necessary to investigate the long-term efficacy of MLD on postoperative edema and pain after TKR.

## Conclusion

Manual Lymphatic Drainage (MLD) does not significantly decrease lymphedema, enhance range of motion, or alleviate pain following Total Knee Replacement (TKR). Consequently, further high-quality research is imperative to accurately evaluate the potential advantages of integrating MLD with standard rehabilitation protocols or other compression techniques in TKR patients.

### Electronic supplementary material

Below is the link to the electronic supplementary material.

Supplementary File 1: Searching strategy for Each Database

Supplementary File 2: Grade evidence for the outcomes

## Data Availability

All data and materials are contained within the manuscript.

## Abbreviations

TKR: Total knee replacement
MLD: Manual lymphatic drainage
ROM: Range of motion
VAS: Visual analog scale
NRS: Numerical Rating Scale
FIM: Functional independence measurement
TKS: Tampa Kinesiophobia Scale
KOOS: Knee Injury and Osteoarthritis Outcome Score
NHP: Nottingham Health Profile
BIS: Bioimpedance Spectroscopy
WOMAC: Western Ontario and McMaster Universities Osteoarthritis Index
KSS: Knee Society Score
