# Supplementary material for: Effects of manual lymphatic drainage on total knee replacement: a systematic review and meta-analysis of randomized controlled trials
Source: BMC Musculoskelet Disord. 2024 Jan 2;25:30. doi: 10.1186/s12891-023-07153-8 (PMC10763325; doi:10.1186/s12891-023-07153-8)
Supplement: Supplementary file 1 — Searching strategy for Each Database [file 12891_2023_7153_MOESM1_ESM.docx]

**Supplementary File 1** Searching strategy for Each Database (Publication date to 2023.07 )

Database No. Searching term Items found

PubMed #1 "Arthroplasty, Replacement, Knee"[Mesh] 30954

#2 knee arthroplasty [All Fields] 46432

#3 total knee arthroplasty [All Fields] 43202

#4 total knee replacement [All Fields] 38520

#5 knee replacement arthroplasty [All Fields] 35656

#6 knee replacement arthroplasties [All Fields] 35275

#7 #1 OR #2 OR #3 OR #4 OR #5 OR #6 49316

#8 "Manual Lymphatic Drainage" [Mesh] 97

#9 manual lymphatic drainage [All Fields] 495

#10 manual lymph drainage [All Fields] 630

#11 decongestive lymphatic therapy[All Fields] 40

#12 Foldi method[All Fields] 176

#13 Vodder method [All Fields] 20

#14 lymphatic drainage massage [All Fields] 544

#15 drainage massage [All Fields] 22

#16 #8 OR #9 OR #10 OR #11 OR #12 OR #13 OR #14 OR #15 883

#17 (randomized controlled trial[pt] OR controlled clinical trial[pt]

OR randomized[tiab] OR placebo[tiab] OR drug therapy[sh] OR randomly[tiab]

OR trial[tiab] OR groups[tiab]) NOT (animals[mh] NOT humans[mh])

#18 #7 AND #16 AND #17 6

Embase #1 'total knee arthroplasty'/exp OR 'total knee arthroplasty' 42246

#2 'manual lymphatic drainage'/exp OR 'manual lymphatic drainage' 909

#3 'crossover procedure':de OR 'double-blind procedure':de

OR 'randomized controlled trial':de OR 'single-blind

procedure':de OR random*:de,ab,ti OR factorial*:de,ab,ti

OR crossover*:de,ab,ti OR ((cross NEXT/1 over*):de,ab,ti) 3104894

OR placebo*:de,ab,ti OR ((doubl* NEAR/1 blind*):de,ab,ti)

OR ((singl* NEAR/1 blind*):de,ab,ti) OR assign*:de,ab,ti

OR allocat*:de,ab,ti OR volunteer*:de,ab,ti

#4 #1 AND #2 AND #3 14

Cochrane #1 MeSH descriptor Arthroplasty, Replacement, Knee explode all trees 3372

Library #2 total knee arthroplasties:ti,ab,kw(Word variations have been searched) 8002

Library #3 total knee replacement:ti,ab,kw(Word variations have been searched) 6196

Library #4 total knee joint prostheses:ti,ab,kw(Word variations have been searched) 193

Library #5 #1 OR #2 OR #3 OR #4 6438

Library #6 MeSH descriptor Manual Lymphatic Drainage explode all trees 25

Library #7 manual lymphatic drainage:ti,ab,kw(Word variations have been searched) 347

Library #8 manual lymph drainage:ti,ab,kw(Word variations have been searched) 170

Library #9 lymphatic drainage massage:ti,ab,kw(Word variations have been searched) 118

Library #10 drainage massage:ti,ab,kw(Word variations have been searched) 173

Library #11 #6 OR #7 OR #8 OR #9 OR #10 448

Library #12 #5 AND #11 11

Web of TS=( (MLD OR manual lymphatic drainage OR " lymphatic drainage massage"

Science OR "drainage massage " OR "Foldi method" OR "Vodder method" ) AND (TKR

OR TKA OR "total knee replacement" OR " total knee arthroplasty" ) ) 15

Google intitle:(( manual lymphatic drainage OR MLD) AND (total knee replacement OR TKR

Scholar OR TKA)) 77

CNKI intitle:(( manual lymphatic drainage ) AND (total knee replacement) 11

VIPs intitle:(( manual lymphatic drainage ) AND (total knee replacement) 6

WanFang intitle:(( manual lymphatic drainage ) AND (total knee replacement) 10
