# Supplementary material for: Effects of manual lymphatic drainage on total knee replacement: a systematic review and meta-analysis of randomized controlled trials
Source: BMC Musculoskelet Disord. 2024 Jan 2;25:30. doi: 10.1186/s12891-023-07153-8 (PMC10763325; doi:10.1186/s12891-023-07153-8)
Supplement: Supplementary file 2 — Grade evidence for the outcomes [file 12891_2023_7153_MOESM2_ESM.docx]

**Author(s):**

**Question:**MLD compared to NO MLD for TKR

**Setting:**

**Bibliography:**

| **Certainty assessment** | | | | | | | | **№ of patients** | | **Effect** | | **Certainty** | **Importance** |
| --- | --- | --- | --- | --- | --- | --- | --- | --- | --- | --- | --- | --- | --- |
| **№ of studies** | | **Study design** | **Risk of bias** | **Inconsistency** | **Indirectness** | **Imprecision** | **Other considerations** | **Intervention** | **Control** | **Relative (95% CI)** | **Absolute (95% CI)** |  |  |
| **ROM (knee flexion)** | | | | | | | | | | | | | |
| 6 | | randomised trials | serious | not serious | not serious | serious | none | 127 | 129 | - | SMD **0.03 SD higher** (0.22 lower to 0.28 higher) | ⨁⨁◯◯ Low^1,2^ | IMPORTANT |
| **ROM (knee extension)** | | | | | | | | | | | | | |
| 3 | | randomised trials | serious | not serious | not serious | serious | none | 66 | 68 | - | SMD **0.3 SD lower** (0.64 lower to 0.04 higher) | ⨁⨁◯◯ Low^2,4^ | IMPORTANT |
| **VAS** | | | | | | | | | | | | | |
| 5 | | randomised trials | serious | serious | not serious | serious | none | 81 | 81 | - | SMD **0.46 SD lower** (1.01 lower to 0.1 higher) | ⨁◯◯◯ Very low^1,3^ | IMPORTANT |
| **NRS** | | | | | | | | | | | | | |
| 2 | | randomised trials | serious | not serious | not serious | serious | none | 57 | 59 | - | SMD **0.12 SD lower** (0.56 lower to 0.31 higher) | ⨁⨁◯◯ Low^1,5^ | IMPORTANT |
| **Thigh circumference** | | | | | | | | | | | | | |
| 4 | | randomised trials | serious | not serious | not serious | serious | none | 90 | 94 | - | SMD **0.15 SD lower** (0.44 lower to 0.14 higher) | ⨁⨁◯◯ Low^1,6^ | IMPORTANT |
| **Calf circumference** | | | | | | | | | | | | | |
| 3 | | randomised trials | serious | not serious | not serious | serious | none | 70 | 74 | - | SMD **0.04 SD lower** (0.37 lower to 0.29 higher) | ⨁⨁◯◯ Low^1,7^ | IMPORTANT |
| **Ankle circumference** | | | | | | | | | | | | | |
| 3 | | randomised trials | serious | not serious | not serious | serious | none | 70 | 74 | - | SMD **0.06 SD lower** (0.39 lower to 0.26 higher) | ⨁⨁◯◯ Low^1,8^ | IMPORTANT |
| ***The risk in the intervention group** (and its 95% confidence interval) is based on the assumed risk in the comparison group and the **relative effect** of the intervention (and its 95% CI).  **CI:** confidence interval; **SMD:** standardised mean difference | | | | | | | | | | | |  |  |
| **GRADE Working Group grades of evidence** **High certainty:** we are very confident that the true effect lies close to that of the estimate of the effect. **Moderate certainty:** we are moderately confident in the effect estimate: the true effect is likely to be close to the estimate of the effect, but there is a possibility that it is substantially different. **Low certainty:** our confidence in the effect estimate is limited: the true effect may be substantially different from the estimate of the effect. **Very low certainty:** we have very little confidence in the effect estimate: the true effect is likely to be substantially different from the estimate of effect. | | | | | | | | | | | |  |  |
| ^1^ Six studies were with high risk of bias ^2^ I^2^=0.0% ^3^ I^2^=63.7% ^4^ One study was with unclear risk of bias. ^5^ I^2^=28.4% ^6^ I^2^=0.0% ^7^ I^2^=31.8%  ^8^ I^2^=0.0% | | | | | | | | | | | |  |  |

**Supplementary File 2: Grade evidence for the outcomes.**
